# Grazing-Induced Conservative Shift in Water-Use Strategies of Desert Plants: Trait Syndromes from Hydraulic Efficiency to Storage Safety

**DOI:** 10.3390/plants15101487

**Published:** 2026-05-13

**Authors:** Jiatong Wu, Yiwei Tang, Chengzhen Jia, Zhiyong Li, Huamin Liu, Lixin Wang, Yang Wang, Lei Dong, Cunzhu Liang, Jinghui Zhang

**Affiliations:** 1 School of Ecology and Environment, Inner Mongolia University, Hohhot 010021, China; 0232121552@mail.imu.edu.cn (J.W.); liuhmimu@aliyun.com (H.L.); lxwimu@foxmail.com (L.W.); bilcz@imu.edu.cn (C.L.); 2 Ministry of Education Key Laboratory of Ecology and Resource Use of the Mongolian Plateau, Inner Mongolia Key Laboratory of Grassland Ecology, Candidate State Key Laboratory of Ministry of Science and Technology, Observation and Research Station for the Typical Steppe Ecosystem of the Ministry of Education, Hohhot 010021, China; 3 Inner Mongolia Institute of Meteorological Sciences, Hohhot 010051, China; 4 College of Life Science and Technology, Inner Mongolia Normal University, Hohhot 010022, China; 5 Institute of Water Resources for Pastoral Areas, Ministry of Water Resources, Hohhot 010020, China

**Keywords:** leaf hydraulic traits, grazing, desert plants, leaf economic spectrum, community-weighted mean (CWM), water-use strategy

## Abstract

Grazing is a pervasive disturbance in arid ecosystems, but its effects on community-level coordination of plant hydraulic and economic traits remain poorly understood. Here, we investigated how long-term grazing alters community-weighted mean hydraulic and leaf economic traits in a desert steppe of Inner Mongolia, and how these shifts affect aboveground biomass (AGB) and water-use efficiency (WUE). Grazing drove a coordinated conservative shift in community hydraulic traits, including more negative osmotic potential at turgor loss point (ψ_tlp_), increased cell wall rigidity (ε), and reduced leaf hydraulic conductance (K_leaf_). Grazing also restructured trait–function relationships: under grazing, AGB was positively correlated with dehydration tolerance rather than transport efficiency, and WUE was tightly coupled with osmotic adjustment. Variance partitioning showed that hydraulic traits explained 57.4% of AGB variation under grazing, whereas economic traits dominated in the control site (74.5%). Our findings demonstrate that long-term grazing imposes a fundamental reorganization of community-level trait coordination, driving a transition from an efficiency-oriented to a safety-oriented strategy, and highlight the central role of hydraulic traits in mediating ecosystem function under combined stress.

## 1. Introduction

Desert ecosystems cover approximately 41% of the global terrestrial area and are indispensable components of the Earth system [1]. Characterized by an arid climate, scarce and highly variable precipitation, and frequent extreme events, these ecosystems support sparse vegetation, poor soils, and low biodiversity [2]. In this harsh environment, water is the primary limiting factor for plant survival, growth, and reproduction. Long-term natural selection, compounded by anthropogenic disturbances like grazing, has driven desert plants to evolve a suite of unique morphological, structural, and functional traits to cope with the dual challenges of drought and herbivory [3]. Plant functional traits, serving as a bridge linking individual physiological processes to community structure and ecosystem function, have become a key entry point for understanding ecosystem responses to global change [4,5].

Grazing is a primary land-use pressure in arid and semi-arid grasslands worldwide, and its effects on ecosystem functioning extend beyond simple biomass removal [6,7]. In desert steppes, long-term overgrazing compacts soil, reduces water infiltration, and exacerbates soil water deficit, imposing additional stress on plant communities already limited by low precipitation [8,9]. Consequently, plants in grazed desert grasslands are frequently subjected to the combined stress of defoliation and drought, which imposes strong selective pressures on both species composition and functional trait expression [10,11]. A global synthesis has shown that grazing consistently favors species with stress-tolerant strategies, including more conservative resource use [12]. Among hydraulic traits, the turgor loss point (ψ_tlp_) is a robust predictor of drought tolerance, with more negative values indicating greater ability to maintain cellular function under water deficit [13,14]. Species capable of osmotic adjustment or modifying cell wall rigidity can better sustain physiological activity under combined grazing and drought stress [15,16].

In recent years, research on plant functional traits has achieved milestones in standardized measurement [17,18] and global data integration [19]. The “Leaf Economics Spectrum” (LES) theory, centered on specific leaf area (SLA) and leaf nutrient content, reveals a global trade-off in plant resource acquisition and conservation strategies, spanning from “fast investment–fast return” to “slow investment–slow return” [20]. This theory has greatly advanced our macro-scale understanding of plant adaptation. However, the LES primarily reflects carbon and nutrient economics, whereas in arid ecosystems, water economy often plays a more decisive role.

In this context, plant hydraulic traits have gained increasing attention [21]. These traits are directly linked to the entire process of plant water transport and use, constituting a key physiological dimension determining drought tolerance, productivity, and survival [22]. Key traits such as leaf hydraulic conductance (K_leaf_, reflecting transport efficiency), water potential at turgor loss point (ψ_tlp_, reflecting drought tolerance), leaf water capacitance (C_leaf_, reflecting storage capacity), and the bulk modulus of elasticity (ε, reflecting cell wall rigidity) together form the “hydraulic toolbox” for coping with water stress [13]. Studies show that under drought, plants often enhance water retention by lowering ψ_tlp_ (osmotic adjustment) and increasing ε, but often at the cost of reduced K_leaf_, reflecting the classic trade-off between hydraulic safety and efficiency [23]. The adjustment of these traits occurs not only at the species level but also at the community level through changes in species composition, influencing ecosystem-level water-use strategies and productivity.

Although the variation in plant hydraulic traits along natural water gradients is relatively well understood [22,24], how grazing—a widespread and intense anthropogenic disturbance—shapes these traits, particularly their coordination with leaf economic traits, remains poorly understood. Grazing directly alters plant population structure through selective foraging and trampling, and indirectly affects soil properties and microclimate, creating a unique “bio-physical” compound stress environment [12]. This challenges plant water acquisition (e.g., through soil compaction) while also influencing resource allocation by altering light and nutrient availability. Sporadic evidence suggests that grazing may induce a shift towards more conservative hydraulic strategies, such as reduced K_leaf_ and ψ_tlp_ [14], or altered leaf structure, such as increased leaf dry matter content (LDMC) [25]. More importantly, under intense stress, plant water-use strategies and resource investment strategies may become tightly coupled [26,27]. For example, high nitrogen content may coordinate with high hydraulic conductance to support rapid growth, while high structural investment (high LDMC) may be associated with strong drought tolerance (more negative ψ_tlp_). However, it is unclear how this network of trait associations is reconfigured under grazing disturbance, what the underlying mechanisms are, and what the cascading effects are on ecosystem functions like aboveground biomass (AGB) and water-use efficiency (WUE). Furthermore, most studies remain at the species level, while interspecific differences and competitive interactions may amplify or offset individual responses at the community level. Therefore, employing community functional metrics such as the community-weighted mean (CWM) is crucial for predicting ecosystem-level responses [28].

To address these knowledge gaps, this study focuses on a typical desert steppe ecosystem in Inner Mongolia under long-term grazing pressure. We aim to answer the following questions: (1) How does grazing affect the expression and coordination of key hydraulic traits in desert plant communities? (2) Under grazing stress, how are hydraulic traits associated with leaf economic traits? Does this support the hypothesis of tight trait coupling under compound stress? (3) How do shifts in community-level hydraulic traits affect key ecosystem functions, including AGB production, grazing response, and WUE? By answering these questions, this study seeks to deepen our theoretical understanding of how arid plants adapt to multiple stresses, particularly the community-level manifestation of trait co-evolution. Practically, it aims to provide a scientific basis for assessing grazing effects and formulating trait-based sustainable management strategies for desert grasslands.

## 2. Results

### 2.1. Effects of Grazing on Community-Level Plant Hydraulic Traits

**Figure 1:**
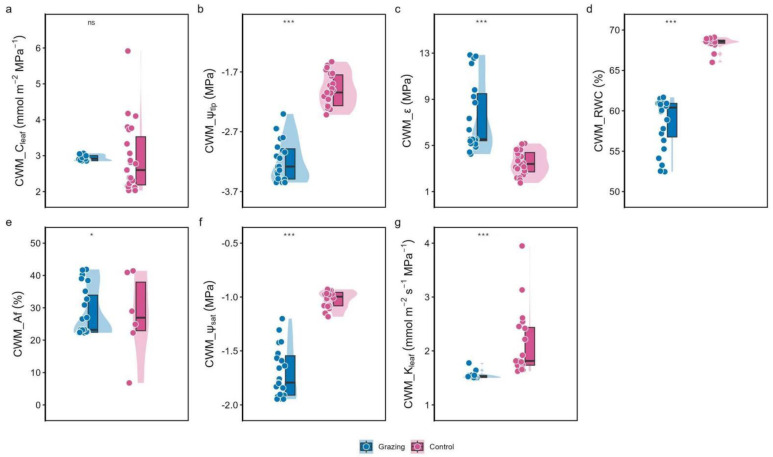
Effects of grazing on community-weighted mean (CWM) hydraulic traits. (**a**) CWM of leaf water capacitance (C_leaf_); (**b**) CWM of osmotic potential at turgor loss point (ψ_tlp_); (**c**) CWM of bulk modulus of elasticity (ε); (**d**) CWM of relative water content at turgor loss point (RWC_tlp_); (**e**) CWM of apoplastic fraction (Af); (**f**) CWM of saturated osmotic potential (ψ_sat_); (**g**) CWM of leaf hydraulic conductance (K_leaf_). Error bars represent standard error (n = 3). *, **, and *** indicate significant differences at *p* < 0.05, *p* < 0.01, and *p* < 0.001, respectively, “ns” (not significant) indicates *p* ≥ 0.05. Based on one-way ANOVA or Kruskal–Wallis test.

Grazing had a highly significant (*p* < 0.001) effect on the community-weighted mean (CWM) of six out of the seven hydraulic traits measured; only CWM_C_leaf_ was not significantly affected. Compared to the enclosed control, the grazing site exhibited significantly lower CWM_ψ_tlp_ and CWM_ψ_sat_, but significantly higher CWM_ε and CWM_Af. CWM_RWC_tlp_ and CWM_K_leaf_ were also significantly lower under grazing (Figure 1).

### 2.2. Relationships Between Community-Level Hydraulic and Economic Traits

**Figure 2:**
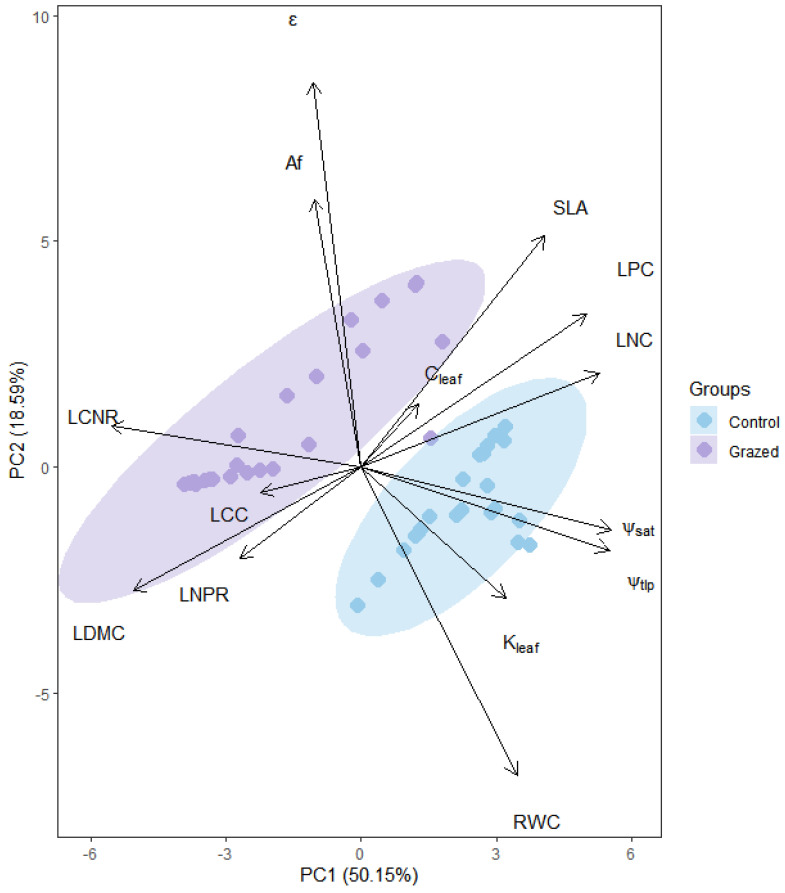
Principal Component Analysis (PCA) of community-weighted mean (CWM) hydraulic and economic traits for communities under enclosed (Control) and grazing management.

Principal Component Analysis (PCA) revealed a clear separation between the functional trait profiles of grazing and enclosed communities (Figure 2). The first two principal components (PCs) explained 68.7% of the total variance (PC1: 50.2%; PC2: 18.5%). PC1 was positively associated with CWM_K_leaf_, CWM_ψ_tlp_, CWM_ψ_sat_, CWM_RWC_tlp_, CWM_SLA, and CWM_LCC. Control communities scored higher on this axis. PC2 was positively associated with CWM_C_leaf_ and CWM_Af and negatively with CWM_RWC_tlp_. Grazing communities scored higher on PC2. This pattern suggests grazing drives a shift in community function from a strategy prioritizing efficient water transport and resource acquisition towards one emphasizing greater water storage capacity and reliance on apoplastic water pathways.

### 2.3. Relationships Among Aboveground Biomass, Grazing Response, and Community-Level Hydraulic Traits

**Figure 3:**
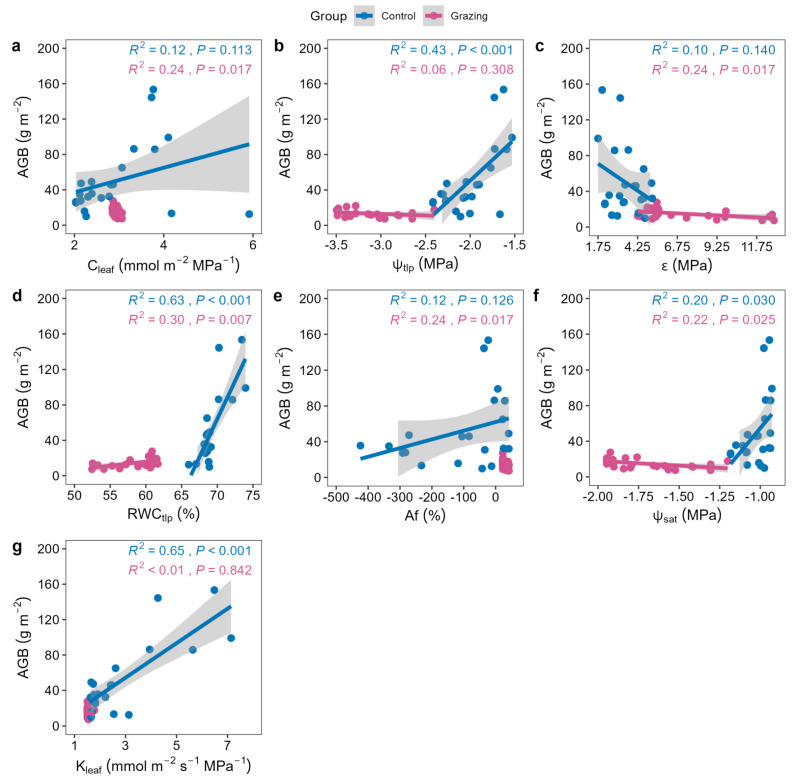
Scatter plots of aboveground biomass (AGB) and community-weighted mean (CWM) hydraulic traits at the community level. Each point represents one quadrat (n = 27 per treatment). (**a**) CWM of leaf water capacitance (C_leaf_); (**b**) CWM of osmotic potential at turgor loss point (ψ_tlp_); (**c**) CWM of bulk modulus of elasticity (ε); (**d**) CWM of relative water content at turgor loss point (RWC_tlp_); (**e**) CWM of apoplastic fraction (Af); (**f**) CWM of saturated osmotic potential (ψ_sat_); (**g**) CWM of leaf hydraulic conductance (K_leaf_).

In the grazing site, AGB showed significant negative correlations with CWM_C_leaf_, CWM_ψ_tlp_, CWM_ε, CWM_Af, and CWM_ψ_sat_, but a significant positive correlation with CWM_RWC_tlp_. No significant correlation was found with CWM_K_leaf_ (Figure 3). In contrast, in the control site, AGB was significantly positively correlated with CWM_ψ_tlp_, CWM_RWC_tlp_, and CWM_K_leaf_ (Figure 3).

**Figure 4:**
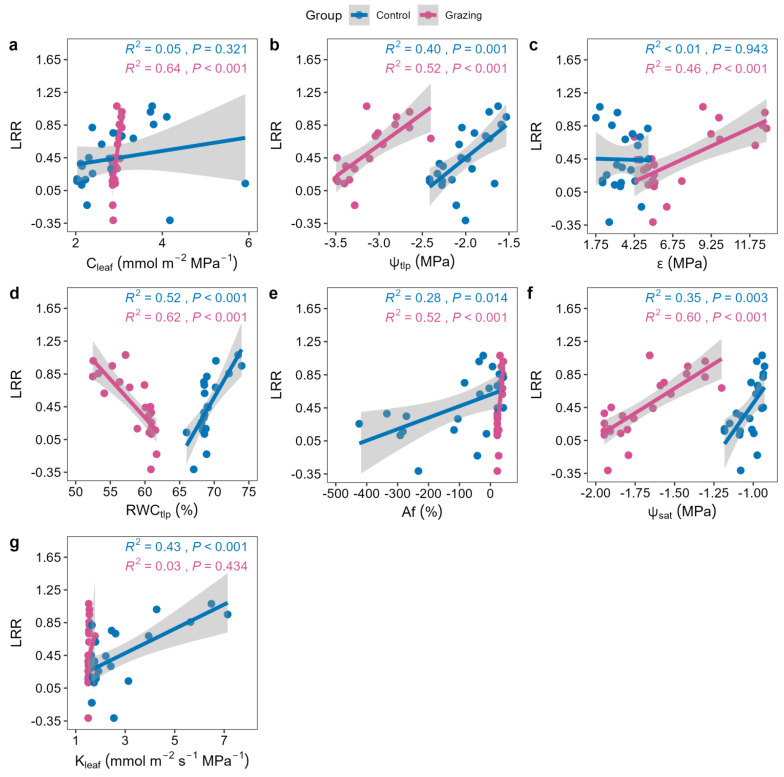
Relationships between the grazing response index (LRR) and community-weighted mean (CWM) hydraulic traits. Each point represents one quadrat (n = 27 per treatment). (**a**) CWM of leaf water capacitance (C_leaf_); (**b**) CWM of osmotic potential at turgor loss point (ψ_tlp_); (**c**) CWM of bulk modulus of elasticity (ε); (**d**) CWM of relative water content at turgor loss point (RWC_tlp_); (**e**) CWM of apoplastic fraction (Af); (**f**) CWM of saturated osmotic potential (ψ_sat_); (**g**) CWM of leaf hydraulic conductance (K_leaf_).

In the grazing site, the grazing response index (LRR) showed significant positive correlations with CWM_C_leaf_, CWM_ψ_tlp_, CWM_ε, CWM_Af, and CWM_ψ_sat_, and a significant negative correlation with CWM_RWC_tlp_ (Figure 4). In the control site, LRR was only significantly positively correlated with CWM_ψ_tlp_, CWM_RWC_tlp_, CWM_Af, CWM_ψ_sat_, and CWM_K_leaf_ (Figure 4).

**Figure 5:**
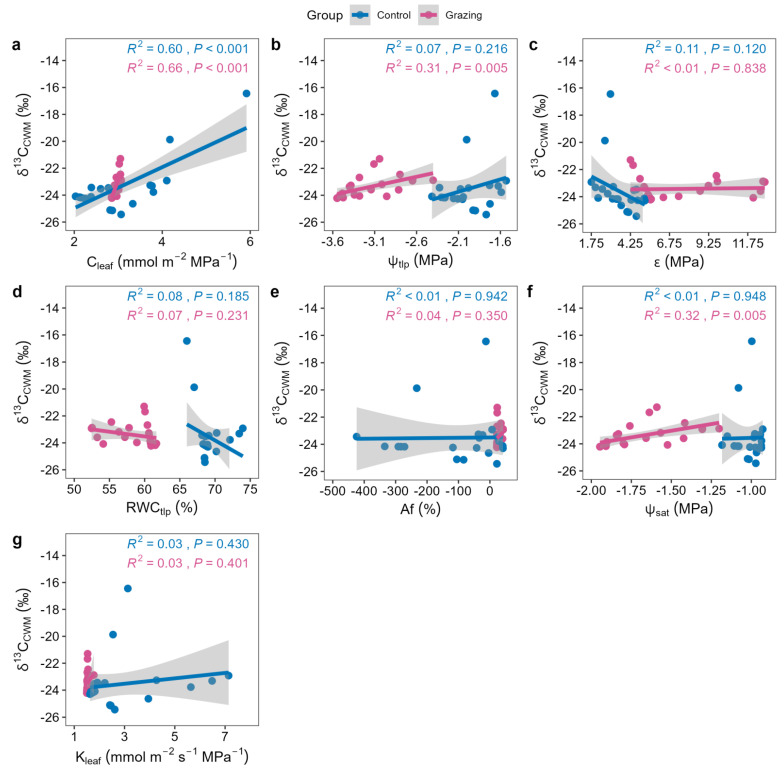
Relationships between leaf carbon isotope composition (δ^13^C, a proxy for intrinsic water-use efficiency) and community-weighted mean (CWM) hydraulic traits. Each point represents one quadrat (n = 27 per treatment). (**a**) CWM of leaf water capacitance (C_leaf_); (**b**) CWM of osmotic potential at turgor loss point (ψ_tlp_); (**c**) CWM of bulk modulus of elasticity (ε); (**d**) CWM of relative water content at turgor loss point (RWC_tlp_); (**e**) CWM of apoplastic fraction (Af); (**f**) CWM of saturated osmotic potential (ψ_sat_); (**g**) CWM of leaf hydraulic conductance (K_leaf_).

Leaf carbon isotope composition (δ^13^C), our proxy for water-use efficiency, was significantly positively correlated with CWM_C_leaf_, CWM_ψ_tlp_, and CWM_ψ_sat_ in the grazing site (Figure 5). In the control site, δ^13^C was only weakly and positively correlated with CWM_C_leaf_ (Figure 5).

### 2.4. Relative Contributions of Hydraulic and Economic Traits to AGB, WUE, and Grazing Response

**Figure 6:**
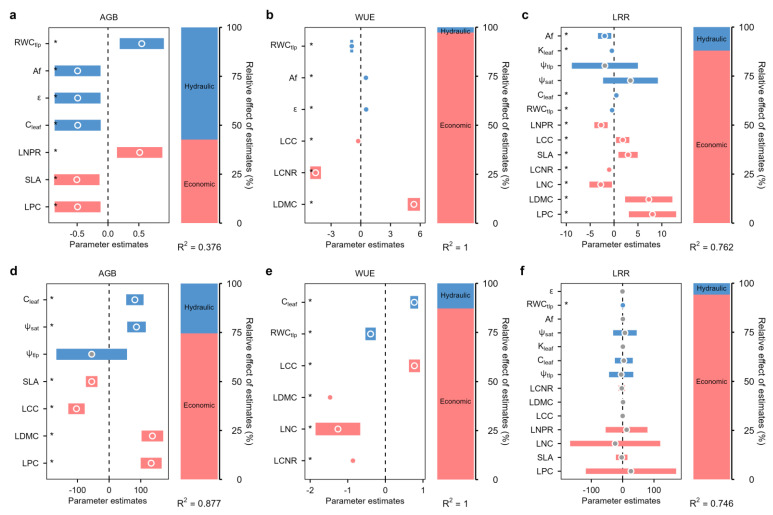
Effects of hydraulic traits and economic traits on aboveground biomass (AGB), water-use efficiency (δ^13^C-derived WUE), and grazing response index (LRR). (**a**–**c**) Grazing site. (**d**–**f**) Control site. On the left, points and shades represent the standardized regression coefficients of model predictors and 95% confidence intervals, respectively. Confidence intervals not overlapping with the dashed line (x = 0) indicate statistical significance (*p* < 0.05). Solid symbols indicate statistical significance (*p* < 0.05), hollow symbols no statistical significance (*p* > 0.05). * denotes statistical significance at the *p* < 0.05 level. On the right, the relative importance of each predictor variable type (expressed as the percentage of explained variance) and the R^2^ of the models are shown.

Overall, plant economic traits exhibited a significant contribution to the variations in AGB, LRR and WUE under both grazing and control conditions. In the grazing site, economic traits explained the most variance in δ^13^C-derived WUE and LRR (97.3% and 88.0%, respectively), while hydraulic traits contributed relatively more to AGB variation, at 57.4% (Figure 6a–c). However, in control site, economic traits were selected as the best predictors of aboveground biomass and grazing response, explaining 74.5% of the variation in AGB, 87.2% of that in WUE, and 94.2% of that in LRR, respectively (Figure 6d–f).

## 3. Discussion

### 3.1. Multi-Scale Effects of Grazing on Plant Hydraulic Traits: From Species Specificity to Community-Level Functional Integration

The impact of grazing on plant hydraulic traits exhibits a pronounced scale effect. While responses vary among dominant species at the individual level, reflecting species-specific adaptation strategies, a clear and consistent adaptive pattern emerges at the community level when integrated via biomass-weighted community-weighted mean (CWM) traits. Our results demonstrate that long-term grazing drives a systematic and coordinated “conservative” shift in the hydraulic trait suite of the desert steppe plant community (Figure 1).

The community enhances “water conservation” and “stress resistance” mechanisms in response to grazing pressure. The significantly lower CWM_ψ_tlp_ and CWM_ψ_sat_ under grazing indicate that community as a whole appears to employ active osmotic adjustment, potentially involving the accumulation of compatible solutes such as proline and glycine betaine, though this hypothesis requires direct biochemical validation in future studies. This allows maintenance of basic physiological activity and turgor at lower tissue water potentials [13], a core physiological mechanism for coping with drought stress [15]. Concurrently, the significantly higher CWM_ε (Figure 1) suggests increased mechanical rigidity of plant cell walls. This structural reinforcement not only reduces the risk of cell collapse during dehydration, maintaining tissue integrity, but also directly enhances leaf resistance to physical damage from trampling [21], exemplifying an integrated adaptation to the compound stress of drought and mechanical disturbance.

The community’s water transport strategy shifts towards “safety-first.” Grazing led to significantly lower CWM_K_leaf_ and higher CWM_A_f_. Reduced K_leaf_ signifies a decline in overall community water transport efficiency, presenting a classic trade-off where “hydraulic efficiency” is sacrificed for “hydraulic safety” [23]. The increased A_f_ indicates a greater community-level reliance on apoplastic pathways (e.g., cell walls) for water movement. Compared to xylem conduits (symplastic pathway), which are prone to embolism, apoplastic pathways are more robust in the face of tissue damage and water potential fluctuations [29]. This strategic shift from efficient-but-vulnerable symplastic transport to less efficient-but-robust apoplastic transport is a key adaptation to potential hydraulic architecture disruption caused by grazing.

The coordinated changes in the above traits lead to an overall conservative water-use strategy. The integrated outcome is manifested in the significantly lower CWM_RWC_tlp_ under grazing (Figure 1). This means the plant community loses turgor at a lower leaf relative water content under grazing disturbance. This is not merely functional decline but an active “water economy” strategy: by increasing the tolerance threshold for wilting, plants can substantially reduce stomatal conductance and transpirational water loss earlier during water stress, thereby maximizing water-use efficiency to extend survival under the dual pressures of drought and herbivory [24]. The PCA results visually support this conclusion (Figure 2): the functional trait distribution of grazing communities clearly diverges from that of control communities, clustering towards the positive end of PC2, which represents high water storage (CWM_C_leaf_) and apoplastic transport (CWM_A_f_). This marks a holistic transition from a “high-efficiency transport–fast growth” strategy to a “safe storage–conservative survival” strategy.

### 3.2. Coupling of Hydraulic and Economic Traits and Community Strategy Remodeling Under Grazing Stress

Our study found that grazing not only alters individual traits but also profoundly reshapes the association network between hydraulic and leaf economic traits, providing strong support for the hypothesis that plant multi-functional trait strategies become tightly coupled under intense environmental stress. The clear separation between grazing and control communities in the PCA (Figure 2) is a direct manifestation of this trait relationship restructuring in the community functional space.

Intriguingly, grazing simultaneously strengthened two contrasting trait syndromes within the community. On one hand, the positive correlation between specific leaf area (SLA) and leaf hydraulic conductance (K_leaf_) was enhanced under grazing. This suggests that even in a stressed environment, a subset of plants maintains a “fast investment–return” strategy. By combining higher SLA (implying thinner leaves and higher photosynthetic potential) with higher K_leaf_ (ensuring water supply), they may attempt to gain a growth advantage in resource-rich microsites created by grazing, such as canopy openings or nutrient patches from dung deposition [20].

On the other hand, and more dominantly, we observed a strong synergy within the “conservative” strategy. High leaf dry matter content (LDMC) was significantly associated with more negative ψ_sat_ and higher ε. This clearly delineates an integrated adaptive pathway: plants invest more carbon resources into cell wall structural components (high LDMC), constructing thicker, more rigid cell walls (high ε), while simultaneously enhancing osmotic adjustment (more negative ψ_sat_). This suite of traits collectively serves to maximize hydraulic safety and tissue stress resistance [30], enabling plants to cope with frequent dehydration and physical damage. This aligns with the resource availability hypothesis, which posits that in stressful environments, reduced growth rates increase the relative benefit of investment in defense and maintenance, leading to an evolutionary shift towards conservative strategies [31].

The positive correlation between leaf nitrogen content (LNC) and K_leaf_ under grazing reveals another facet of nutrient–water co-utilization. Grazing may alter the resource environment through canopy opening (increased light) and dung deposition (increased nitrogen input). Under such conditions, some plants may adopt a “high nitrogen metabolism–high water transport” strategy, maintaining higher nitrogen content to support higher physiological activity, which in turn requires higher hydraulic conductance to ensure water supply and avoid accumulation of nitrogen metabolites [32]. This stands in sharp contrast to the decoupling of hydraulic and economic traits often observed in water-sufficient ecosystems [27], underscoring that environmental stress intensity is a key selective pressure driving trait co-evolution [33]. Under the compound stress of aridity and grazing, water becomes the central hub linking carbon and nitrogen resource use, forcing a tight integration and trade-off among various plant functional traits around the water economy.

### 3.3. Cascading Effects of Community Hydraulic Trait Shifts on Ecosystem Functions

The “conservative” shift in community hydraulic traits is not an isolated physiological response but triggers cascading changes in ecosystem functions. Our results reveal a profound restructuring of trait–function relationships under grazing disturbance. In the grazing site, AGB was positively correlated with RWC_tlp_ (representing dehydration tolerance) but negatively correlated with C_leaf_ (water storage capacity) and ψ_sat_ (water status at full hydration) (Figure 3). This seemingly counterintuitive pattern reveals that under chronic stress, community biomass production no longer depends on the potential for rapid growth at high water potentials (which would require high C_leaf_ and ψ_sat_), but rather on the plants’ ability to maintain cell turgor and continued physiological activity during drought periods. The baseline for biomass accumulation thus shifts from “growth potential” to “survival capacity.” This is fundamentally different from the “efficiency-driven” pattern in the control site, where AGB was positively correlated with efficient water transport (K_leaf_) and higher water potential (ψ_tlp_) (Figure 3), reflecting a paradigm shift from “growth-priority” to “survival-priority” strategies [23].

The grazing response index (LRR) reveals complex relationships between trait adaptation and ecosystem resistance. We found LRR to be positively correlated with more conservative hydraulic traits (e.g., more negative ψ_tlp_, higher ε) (Figure 4). This positive correlation suggests that species with higher hydraulic safety and greater structural investment (‘conservative’ types) experience a greater proportional loss of aboveground biomass under grazing. A plausible hypothesis to explain this pattern—and the resulting community-level conservative shift—is that conservative plants may possess strong survival capacities (e.g., developed root systems, dormant bud banks) that allow them to tolerate higher aboveground tissue loss. Under long-term grazing selection, such species could persist via this tolerance strategy and, over time, increase their dominance within the community, even though each individual loses more biomass in the short term. However, we did not measure belowground or regenerative traits (e.g., root biomass, bud bank size), and thus this interpretation remains a hypothesis requiring direct testing in future studies. Nevertheless, the positive correlation between LRR and conservative traits clearly indicates that short-term biomass loss and long-term community trait shifts are linked through species turnover. LRR reflects instantaneous biomass loss (system sensitivity), while CWM traits reflect long-term adaptive outcomes (system resistance). Their positive correlation highlights the complex interaction between short-term functional response and long-term structural adaptation, and the mechanism by which ecosystems achieve functional stability through species turnover.

Improved water-use efficiency is explicitly linked to osmotic adjustment. In the grazing site, WUE was tightly and positively correlated with osmotic adjustment-related traits (ψ_tlp_, ψ_sat_) (Figure 5), confirming that active lowering of osmotic potential to enhance WUE is a core internal physiological mechanism for coping with grazing-aggravated water stress [16]. The community-wide shift towards more negative osmotic potentials directly results in higher WUE. In the undisturbed control site, WUE was only weakly correlated with C_leaf_ and decoupled from other hydraulic traits (Figure 5), suggesting that under relatively stable water conditions, WUE may be regulated by a combination of factors like stomatal behavior and root distribution, leading to more diverse strategies [34]. This further indicates that grazing, as a strong selective pressure, simplifies the functional strategies of plant communities, forcing them to converge on a high-efficiency water-use mode centered on enhanced osmotic adjustment.

### 3.4. The Shifting Relevance of Hydraulic Versus Economic Traits in Explaining Ecosystem Function

Beyond the specific trait–function relationships, our variance partitioning analysis (Figure 6) reveals a fundamental shift in the relative importance of hydraulic and economic traits for ecosystem functioning under grazing. In the control site, economic traits overwhelmingly dominated the explained variance in AGB (74.5%), WUE (87.2%), and LRR (94.2%). This aligns with the classic leaf economics spectrum perspective, where resource allocation strategies (e.g., SLA, LDMC, LNC) are primary drivers of productivity and resource use in relatively resource-rich environments [20].

However, under grazing stress, this pattern changed dramatically. While economic traits remained important for WUE and LRR, hydraulic traits emerged as substantial contributors, explaining 57.4% of the variation in AGB. This indicates that when water becomes the primary limiting factor—exacerbated by grazing-induced soil compaction and reduced infiltration—the community’s productivity becomes increasingly constrained by hydraulic characteristics such as transport efficiency (K_leaf_), dehydration tolerance (ψ_tlp_, RWC_tlp_), and water storage capacity (C_leaf_). The ability to move, store, and conserve water supersedes the ability to acquire carbon and nutrients as the key determinant of biomass accumulation.

This shift in explanatory power underscores a critical insight: environmental stress reorders the hierarchy of plant functional traits governing ecosystem function. In benign conditions, economic traits (carbon–nutrient economics) dominate; under stress, hydraulic traits (water economics) take precedence. This finding not only supports our central thesis that grazing induces a conservative shift in water-use strategy but also provides a mechanistic explanation for why hydraulic traits become increasingly important predictors of ecosystem function in arid and disturbed environments.

### 3.5. Conclusions and Perspectives

This study reveals, from a community-scale perspective, that long-term grazing drives a coordinated shift in both hydraulic and economic traits of desert steppe plant communities, collectively pointing towards a high-safety, low-efficiency “conservative” survival strategy. This multi-dimensional trait co-evolution is a core mechanism by which plant communities adapt to the compound stress of aridity and grazing, ultimately reshaping system productivity patterns and water-use efficiency by altering the relationships between traits and ecosystem functions. Notably, we demonstrate that grazing not only shifts trait values but fundamentally reorders the relative importance of hydraulic versus economic traits in governing ecosystem function, with hydraulic traits becoming primary determinants of productivity under stress. Our findings emphasize that assessing the ecological effects of grazing and managing arid grasslands must adopt an integrated perspective focusing on combinations of plant functional traits and their interrelationships, with particular attention to the water economy.

Future research could deepen understanding in the following directions: (1) integrating root hydraulic traits with soil moisture dynamics monitoring to construct a complete picture of adaptation along the “soil–plant–atmosphere” continuum; (2) conducting controlled experiments with multiple grazing intensity gradients to disentangle the interactive effects of grazing intensity and drought stress on trait plasticity and genetic adaptation; (3) linking functional trait responses to higher-level ecosystem services such as stability and carbon sequestration to develop trait-based ecological prediction models, providing more precise theoretical tools for the adaptive management of desert grasslands.

## 4. Materials and Methods

### 4.1. Study Area and Experimental Design

**Figure 7:**
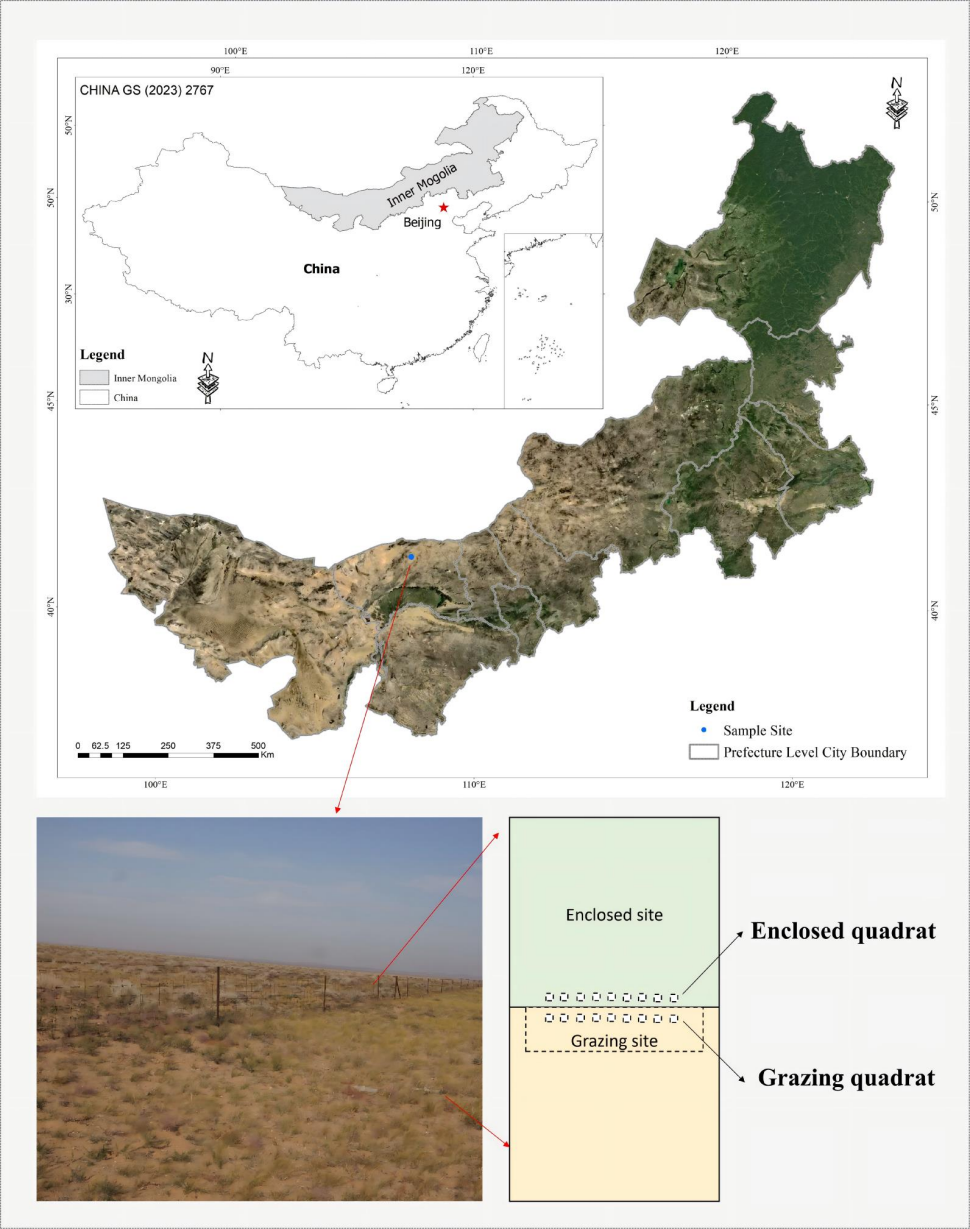
Study area location and experimental design.

The study was conducted in a desert steppe in Urad Middle Banner, Bayannur City, Inner Mongolia Autonomous Region, China (41°24′–42°28′ N, 106°16′–109°42′ E; ~1289 m a.s.l., Figure 7). The region experiences a temperate continental arid climate, with a multi-year (1954–2026) mean annual temperature of approximately 5.6 °C and mean annual precipitation of 202.4 mm, mostly concentrated in short-duration summer events. During the study year (2023), the annual mean temperature was 7.6 °C and annual precipitation was 127.9 mm (Table S1). The soil type is primarily brown calcisol (Aridisol). The natural grasslands in the area have a long history of livestock grazing, with grazing banned in some areas since the 1990s.

To compare ecosystem differences under contrasting grazing management histories, we established three spatially independent paired sites across the study region, with each pair separated by several kilometers. Each pair consisted of one long-term fenced enclosure (established in 2002, hereafter “control”) and one adjacent grazing site (hereafter “grazed”) belonging to the same household. All paired sites shared similar soil conditions and topography (Tables S2 and S3).

We acknowledge that because the fenced sites have been fully protected since 2002 while the grazed sites have been continuously grazed for decades, the two site types inherently differ in their grazing history. Therefore, our comparison reflects the integrated effects of long-term management history rather than a pure manipulative grazing treatment, and we do not claim causal attribution of all observed differences to current grazing alone. Instead, this study documents the current ecosystem state under contrasting long-term management regimes, with spatial replication across three independent locations to enhance the robustness of our findings.

Within each grazing pasture, a 0.5 km × 2 km area was fenced as the grazing site (Grazed, G). From June to September each year (grazing season: 4 months), these grazing sites were grazed at a stocking rate of 1.8 sheep per hectare using domestic sheep (Ovis aries) with a mean body weight of approximately 60 kg. Grazing was continuous (24 h per day) throughout the season, with no rotational grazing or rest periods. All three grazing sites were managed by the same household to ensure consistency in management practices. The long-term fenced enclosure sites (control) received no grazing since their establishment in 2002.

At each site (control and grazed), three replicate 100 m × 100 m plots were established along the fenceline (Figure 7). To avoid interference between sampling activities, each plot was subdivided into two halves: one designated for plant community surveys and the other for plant functional trait measurements. Within the community survey half, three 10 m × 10 m shrub quadrats were established. At each corner of every shrub quadrat, one 1 m × 1 m herbaceous quadrat was placed to ensure comprehensive representation of the community structure [35].

### 4.2. Plant Functional Trait Measurements

#### 4.2.1. Leaf Hydraulic Traits

Seven key leaf hydraulic traits were measured: leaf water capacitance (C_leaf_), apoplastic fraction (Af), saturated osmotic potential (ψ_sat_), bulk modulus of elasticity (ε), osmotic potential at turgor loss point (ψ_tlp_), relative water content at turgor loss point (RWC_tlp_), and leaf hydraulic conductance (K_leaf_). For each species per treatment, leaves were collected from a consistent position on the stem and at a defined stage of development mature leaves. Measurements were taken from 15 different individuals per species per treatment, with one replicate per individual.

Parameters (C_leaf_, Af, ψ_sat_, ε, ψ_tlp_, RWC_tlp_) were derived from P-V curves obtained using the bench-drying method [36] following standardized protocols [37]. Briefly, fully expanded, healthy leaves were collected pre-dawn, rehydrated to full turgor, and then allowed to dry on the bench. Leaf mass and water potential were measured periodically until the leaf reached a water potential below −4 MPa. P-V curves were plotted as the inverse of water potential (1/ψ) against relative water content (RWC).

Leaf Hydraulic Conductance: K_leaf_ was measured using the rehydration kinetic method (RKM) following Brodribb & Holbrook (2003) [14]. Leaves were excised under water and allowed to rehydrate for a controlled period (t = 30–300 s). Initial (Ψ_0_) and final (Ψf) leaf water potentials were measured using a pressure chamber. Leaf capacitance (C_leaf_, mmol m^−2^ MPa^−1^) was determined independently from pressure–volume curves. Leaf area (cm^2^) was measured from scanned images and converted to m^2^ (divided by 10,000). K_leaf_ was then calculated as:

K_leaf_ = C_leaf_ × ln(Ψ_0_/Ψ_f_)/t,

with final units of mmol m^−2^ s^−1^ MPa^−1^.

K_leaf_ was measured using the leaf water potential relaxation method [14]. Leaves were collected, rehydrated, and then subjected to a controlled desiccation period. The rate of water potential equilibration upon rehydration was used to calculate K_leaf_, which reflects the efficiency of water transport through the leaf lamina (Table 1).

#### 4.2.2. Leaf Economic Traits

Six dominant plant species, whose combined biomass and cover accounted for over 80% of the total community, were selected for measurement. The dominant species in the study area were *Stipa klemenzii*, *Cleistogenes songorica*, *Allium polyrhizum*, *Caragana brachypoda*, *Caragana spinifera*, and *Krascheninnikovia ceratoides*. Their relative biomass is shown in Table S1. For each species under each treatment, ten healthy, mature individuals were randomly selected, and fully expanded sun leaves were collected. Leaf area (LA) was determined from scanned leaf images (CanoScan LiDE 300 (Canon Inc., Tokyo, Japan)) using ImageJ software. Leaves were oven-dried at 65 °C to constant weight to determine leaf dry weight (LDW). Specific leaf area (SLA) was calculated as LA/LDW. Leaf dry matter content (LDMC) was calculated as LDW/leaf fresh weight (LFW).

Dried and ground leaf material was used to determine leaf carbon content (LCC) and leaf nitrogen content (LNC) using an elemental analyzer (LECO CHN-600) (LECO Corp., St. Joseph, MI, USA). Leaf phosphorus content (LPC) was determined using inductively coupled plasma optical emission spectrometry (ICP-OES). The leaf carbon-to-nitrogen ratio (LCNR) and nitrogen-to-phosphorus ratio (LNPR) were subsequently calculated (Table 2).

### 4.3. Leaf Carbon Isotope Composition (δ^13^C) and Water-Use Efficiency (WUE)

Leaf carbon isotope composition (δ^13^C) was used as a time-integrated proxy for intrinsic water-use efficiency (WUE). Dried and ground leaf samples were analyzed using a coupled system consisting of an elemental analyzer (Vario Micro cube, Elementar Analysensysteme GmbH, Langenselbold, Germany) and an isotope ratio mass spectrometer (Delta V Advantage, Thermo Fisher Scientific, Bremen, Germany). The foliar δ^13^C value was calculated as:

(1) $$\delta^{13}C=\left[\left(\frac{R_{\mathrm{sample}}}{R_{\mathrm{standard}}}\right)-1\right]\times 1000‰$$

where $R_{\mathrm{sample}}$ denotes the ^13^C/^12^C molar ratio in the sample and $R_{\mathrm{standard}}$ denotes the ^13^C/^12^C molar ratio in the Pee Dee Belemnite standard.

Higher δ^13^C values (less negative) indicate greater water-use efficiency, as they reflect lower intercellular CO_2_ concentration resulting from partial stomatal closure under water-limited conditions [38]. Throughout this manuscript, WUE refers to δ^13^C-derived intrinsic water-use efficiency.

### 4.4. Aboveground Biomass (AGB) Measurement

#### Measurement of Aboveground Biomass (AGB)

In the winter of 2022, we placed nine 1 m^3^ cages in each grazing site to facilitate subsequent calculation of plant growth. The cages were moved to new locations each winter to avoid localized over-protection. In August 2023 (the peak growing season), one 1 m × 1 m quadrat was established near each cage within the grazing site, and a corresponding 1 m × 1 m quadrat was established at the matching position within the adjacent enclosed (control) site. For the calculation of the log response ratio (LRR), each pair of grazing and control quadrats was located no more than 3 m from the shared fence.

In total, we established 27 quadrats in the grazing sites (3 grazing sites × 9 quadrats per site) and 27 quadrats in the control sites (3 control sites × 9 quadrats per site), resulting in 27 paired quadrats across the three spatially independent paired sites. All living vascular plants within the quadrats were harvested by clipping to 1 cm stubble height, sorted by species, oven-dried at 105 °C for 20 min, and then dried to a constant weight at 65 °C. AGB was determined by weighing the dried plant material.

For shrubs, we measured the crown diameter (two perpendicular directions) and plant height for each individual in the survey plots. To convert these measurements into biomass, we developed species-specific allometric equations prior to the formal experiment. For each of the three dominant shrub species (*Caragana brachypoda*, *Caragana spinifera*, and *Krascheninnikovia ceratoides*), we destructively sampled 15–20 individuals covering a range of sizes. For each individual, we measured crown diameter and height, then harvested the whole plant, oven-dried it at 65 °C to constant weight, and fitted power-law regressions using crown diameter as the predictor variable. The resulting equations (R^2^ = 0.82–0.94) are provided in Table S5. Shrub biomass in the survey plots was then estimated using these equations.

### 4.5. Data Analysis

Community-Weighted Mean (CWM) Traits: To scale from species-level traits to the community level, the CWM for each functional trait was calculated based on species relative biomass contributions using the FD package in R [39]. For each plot, CWM_trait_ = Σ (p_i_ × trait_i_), where p_i_ is the relative biomass of species *i*, and trait_i_ is its mean trait value.

Grazing Response Index: The log response ratio (LRR) was used to quantify the grazing effect on community aboveground biomass: LRR = ln(AGBcontrol/AGBgrazing). A positive LRR indicates a reduction in biomass due to grazing.

Statistical Analysis: Data normality and homogeneity of variances were tested using the Shapiro–Wilk test and Levene’s test, respectively. For normally distributed data, one-way ANOVA was used to compare trait CWM differences between grazing and control treatments, followed by Tukey’s HSD post hoc test if significant. The non-parametric Kruskal–Wallis test was used for non-normal data. Relationships between traits, and between traits and AGB, LRR, or WUE (δ^13^C), were analyzed using ordinary least squares (OLS) regression. Principal Component Analysis (PCA) was performed on the seven hydraulic and seven economic traits at the community level (CWM values) to reduce dimensionality and reveal major patterns of trait variation and functional differentiation between grazing and control communities. Prior to PCA, all trait variables were centered and scaled (standardized to mean = 0 and SD = 1) to account for differences in units and measurement scales. No missing values were present. The PCA was performed using plot-level CWM values. All statistical analyses were performed in R version 4.3.0 [40]. Statistical significance was set at *p* < 0.05.

## Figures and Tables

**Table 1:** Abbreviations, units, and definitions of measured plant leaf hydraulic traits.

Plant Trait	Abbreviation	Unit	Definition
Leaf water capacitance	C_leaf_	mmol m^−2^ MPa^−1^	Water storage capacity per unit change in water potential.
Leaf hydraulic conductance	K_leaf_	mmol m^−2^ s^−1^ MPa^−1^	Efficiency of water transport through the leaf.
Saturated osmotic potential	ψ_sat_	MPa	Osmotic potential of the symplast when the leaf is fully hydrated.
Apoplastic fraction	Af	%	Proportion of leaf water residing in the apoplast.
Relative water content at turgor loss point	RWC_tlp_	%	Leaf relative water content when turgor pressure reaches zero.
Bulk modulus of elasticity	ε	MPa	Measure of cell wall stiffness (inverse of elasticity).
Osmotic potential at turgor loss point	ψ_tlp_	MPa	Osmotic potential when leaf turgor pressure is lost.

**Table 2:** Abbreviations and units of measured plant leaf economic traits.

Leaf Economic Trait	Abbreviation	Unit
Specific leaf area	SLA	cm^2^ g^−1^
Leaf dry matter content	LDMC	mg g^−1^
Leaf carbon content	LCC	mg g^−1^
Leaf nitrogen content	LNC	mg g^−1^
Leaf phosphorus content	LPC	mg g^−1^
Leaf carbon-to-nitrogen ratio	LCNR	-
Leaf nitrogen-to-phosphorus ratio	LNPR	-

## Data Availability

The original data presented in the study are included in the article; further inquiries can be directed to the corresponding author.

## Supplementary Materials

The following supporting information can be downloaded at: https://www.mdpi.com/article/10.3390/plants15101487/s1. Table S1. Monthly and annual comparison of climatic conditions: multi-year average (1954–2026) vs. study period (2023). Table S2. Comparison of control site in the region of Urat Desert-grassland. Mean and ±1 SE of different parameters are reported along with P-values of one-way ANOVA (measured in 2021). Table S3. Comparison of grazed site in the region of Urat Desert-grassland. Mean and ±1 SE of different parameters are reported along with P-values of one-way ANOVA (measured in 2021). Table S4. The mean relative contribution of dominant species at control sites and grazed sites (tested by *t* test). Table S5. Allometric equations for estimating aboveground biomass (g) of dominant shrub species from crown diameter (CD, cm).
