# Supplementary material for: Grazing-Induced Conservative Shift in Water-Use Strategies of Desert Plants: Trait Syndromes from Hydraulic Efficiency to Storage Safety
Source: Plants (Basel). 2026 May 13;15(10):1487. doi: 10.3390/plants15101487 (PMC13211288; doi:10.3390/plants15101487)
Supplement: Supplementary file 1 [file plants-15-01487-s001.zip › plants-4246460-supplementary.pdf]

Supplementary Information for

Grazing-Induced Conservative Shift in Water-Use Strategies of Desert Plants: Trait Syndromes from Hydraulic Efficiency to Storage Safety

Jiatong Wu [1,2], Yiwei Tang [1,2], Chengzhen Jia [3], Zhiyong Li [1,2], Huamin Liu [1,2], Lixin Wang [1,2], Yang Wang [4], Lei Dong [5], Cunzhu Liang [1,2] and Jinghui Zhang [1,2]*

[1] School of Ecology and Environment, Inner Mongolia University, Hohhot, China;
[2] Ministry of Education Key Laboratory of Ecology and Resource Use of the Mongolian Plateau & Inner Mongolia Key Laboratory of Grassland Ecology and the Candidate State Key Laboratory of Ministry of Science and Technology & Observation and Research Station for the Typical Steppe Ecosystem of the Ministry of Education, Hohhot, China;
[3] Inner Mongolia Institute of Meteorological Sciences, Hohhot, China;
[4] College of Life Science and Technology, Inner Mongolia Normal University, Hohhot, China;
[5] Institute of Water Resources for Pastoral Areas, Ministry of Water Resources, Hohhot, China
* Correspondence author: Jinghui Zhang, Email: jhzhang1001@126.com.

This file includes:

Table. S1 to S5

Table S1. Monthly and annual comparison of climatic conditions: multi-year average (1954–2026) vs. study period (2023)

Month	Mean Temperature (°C)		Precipitation (mm)	
	Multi-year (1954–2026)	2023	Multi-year (1954–2026)	2023
Jan	-13.2	-11.6	1.2	0.4
Feb	-9.5	-4.4	1.1	2.4
Mar	-1.6	2.0	3.0	0.0
Apr	7.3	8.3	6.0	3.0
May	14.8	15.9	12.4	32.4
Jun	20.3	22.3	28.8	4.5
Jul	22.5	23.5	52.1	20.3
Aug	20.7	23.6	57.5	15.1
Sep	14.2	17.3	26.5	42.9
Oct	5.9	9.5	9.5	1.0
Nov	-4.0	-4.2	3.0	1.6
Dec	-11.4	-11.7	1.3	4.3
Annual	5.6	7.6	202.4	127.9

Table S2 Comparison of control site in the region of Urat Desert-grassland. Mean and ±1 SE of different parameters are reported along with P-values of one-way ANOVA (measured in 2021)

Parameter	Control site 1	Control site 2	Control site 3	*p*-Value
pH	8.56±0.13	8.77±0.11	8.52±0.06	0.285
Soil volumetric water content (%)	3.71±0.80	1.33±0.31	6.39±2.46	0.136
Soil organic carbon ($g \cdot kg^{-1}$)	5.39±2.17	4.81±0.96	6.61±0.29	0.663
Soil organic matter ($g \cdot kg^{-1}$)	9.29±3.74	8.30±1.66	11.39±0.51	0.663

Table S3 Comparison of grazed site in the region of Urat Desert-grassland. Mean and ±1 SE of different parameters are reported along with P-values of one-way ANOVA (measured in 2021)

Parameter	Grazed site 1	Grazed site 2	Grazed site 3	*p*-Value
pH	8.57±0.04	8.62±0.05	8.52±0.02	0.204
Soil volumetric water content (%)	3.83±0.75	4.46±0.23	4.92±1.50	0.743
Soil organic carbon ($g\cdot kg^{-1}$)	8.15±0.61	7.25±4.03	5.69±2.24	0.727
Soil organic matter ($g\cdot kg^{-1}$)	14.06±1.06	12.49±6.95	9.81±3.85	0.727

Table S4. The mean relative contribution of dominant species at control sites and grazed sites (tested by t test).

Species	Mean±SE		*p*-Value
	Control Site	Grazed Site	
Stipa klemenzii	25.96 ± 5.23 b	65.97 ± 4.87 a	< 0.001
Cleistogenes songorica	9.58 ± 3.64	11.32 ± 2.41	0.687
Allium polyrhizum	0.23 ± 0.14 b	9.86 ± 2.73 a	< 0.001
Caragana brachypoda	22.45 ± 6.32 a	0.89 ± 0.58 b	< 0.001
Caragana spinifera	9.76 ± 4.88 a	0 b	< 0.01
Krascheninnikovia ceratoides	13.68 ± 5.42 a	2.03 ± 1.76 b	< 0.05
Total	82.66 ± 4.89	90.07 ± 3.25	0.058

Table S5. Allometric equations for estimating aboveground biomass (g) of dominant shrub species from crown diameter (CD, cm).

Species	Equation	R^2	n
Caragana brachypoda	Biomass = $0.32 \times CD^{2.18}$	0.91	18
Caragana spinifera	Biomass = $0.28 \times CD^{2.05}$	0.88	15
Krascheninnikovia ceratoides	Biomass = $0.45 \times CD^{1.96}$	0.94	20

Note: CD is the average of two perpendicular crown diameter measurements (cm). Equations were developed from destructive sampling in the study area prior to the formal experiment.
